# Multiple Myeloma and Diabetes

**DOI:** 10.5402/2011/815013

**Published:** 2011-11-17

**Authors:** Zeinab A. Issa, Mira S. Zantout, Sami T. Azar

**Affiliations:** Division of Endocrinology and Metabolism, Department of Internal Medicine, American University of Beirut-Medical Center, 3 Dag Hammarskjold Plaza, 8th floor, New York, NY 10017, USA

## Abstract

Multiple myeloma is a malignant plasma cell disorder that accounts for approximately 10% of all hematologic cancers. It is characterized by accumulation of clonal plasma cells, predominantly in the bone marrow. The prevalence of type 2 diabetes is increasing; therefore, it is expected that there will be an increase in the diagnosis of multiple myeloma with concomitant diabetes mellitus. The treatment of multiple myeloma and diabetes mellitus is multifaceted. The coexistence of the two conditions in a patient forms a major challenge for physicians.

## 1. Introduction

It is estimated that around 8–18% of cancer patients have diabetes. Diabetes and cancer are two overwhelming conditions for both patients and clinicians. The treatment of diabetes in the presence of cancer is a major challenge for physicians. Maintaining adequate glucose control is a crucial factor in preventing infections in at-risk cancer patients [1]. Multiple myeloma is a fatal neoplasm of the B cell characterized by expansion of malignant plasma cells, mostly in the bone marrow which in return leads to one or more clinical manifestation of bone destruction, hypercalcemia, anemia, and renal insufficiency. The disease accounts for approximately 10% of all hematologic cancers [2].

 Since the prevalence of type 2 diabetes is increasing worldwide, an increase in the diagnosis of MM with concomitant DM is expected. Therefore, physicians treating such patients should be fully aware of the potential effect of MM treatment on glucose metabolism in this population [3]. 

Multiple reports have linked diabetes to increased risk of cancer mainly pancreatic, liver, colon, breast, and endometrial cancer [4]. In a phase 3 Apex trial in patients with relapsed multiple myeloma by Richardson et al., 18% patients had either a baseline glycosylated hemoglobin higher than normal upper level or a history of diabetes [5]. In other reports, the prevalence was between 11% and 22% [6, 7]. 

Is there evidence about a causal relationship? Although results in the literature are contradictory, in a recent study conducted by Khan et al. there was no association between self reported diabetes and multiple myeloma [8], whereas the highest level of postload glucose was associated with risk of mortality from multiple myeloma (HR, 3.06; 95% CI, 1.05–8.93) in another study by Chiu et al. [9].

There have been outstanding improvements over the past decade in the area of initial therapy of newly diagnosed multiple myeloma. Several large trials investigated the role of treatment regimens involving one or more of the most recent medications. [10–18]. Many factors govern the choice of initial therapy for MM. The patient's age, performance status, eligibility for stem cell therapy, and most importantly the presence of disease-related complications as well as other comorbid conditions such as diabetes and obesity are factors to consider before the choice of initial therapy. Introduction of new more efficient treatments, in addition to expansion in the use of high-dose therapy, is a factor that contributed to better prognosis with an effect on diabetes control. Novel agents have been introduced, namely, bortezomib, thalidomide, and lenalidomide. In addition to these three novel agents, other targeted therapies are being investigated in preclinical and clinical studies as well as treatments combining these agents with other novel agents together with traditional drugs that are used commonly. These trials are exhibiting a promising future in the treatment of myeloma. However, the safety and efficacy of combinations integrating these novel agents on diabetes control and complications is not well understood [19].

## 2. Glucose Control in Multiple Myeloma

Dexamethasone- and prednisone-based regimens are part of the conventional and new methods to treat newly diagnosed or recurrent/multiple myeloma, These medications raise blood glucose through increased insulin resistance, gluconeogenesis, glycogenolysis, and decreased insulin production and secretion [20]. Glucocorticoids are frequently used in high doses for a short term during chemotherapy protocol whereas lower doses are also used to prevent chemotherapy-induced nausea and vomiting. 

Dexamethasone was shown to be more harmful to the diabetes profile in a study by Facon et al. where the investigators compared dexamethasone and Prednisone-based regimens with standard melphalan prednisone in newly diagnosed MM patients ineligible for high-dose therapy. The morbidity associated with dexamethasone-based regimens was significantly higher than with melphalan prednisone including severe diabetes [21]. We suggest that patients should be screened for diabetes before starting glucocorticoid treatment and monitored closely. Glucocorticoid-free regimens can be used in patients with diabetes mellitus [22]. Risk factors for glucocorticoid-induced diabetes including obesity, age, family history of diabetes, personal history of gestational diabetes, and high-dose steroids are all prompts for a more stringent screening [23]. Oral hypoglycemic agents can be continued if they seem to be enough for adequate glycemic control; however, patients will frequently need insulin as a an add on therapy. Patients already on insulin will most likely require basal and preprandial doses, up to two to three times their usual dose to adequately control their blood sugar levels [20, 23, 24].

Patients with multiple myeloma may experience nausea and vomiting in addition to poor appetite and thus missed meals which put patients at risk of hypoglycemia. Treating the nausea and vomiting by antiemetics; advising patients to eat small frequent meals and to avoid sweet, salty, or spicy foods since they may aggravate nausea and vomiting will minimize the risk of hypoglycemia in such patients. Additionally, using a short acting secretagogue (nateglinide or repaglinide) instead of a usual sulfonylurea (glimepiride, glipizide, or glyburide) may be a better option for postprandial hyperglycemia to avoid hypoglycemia; moreover, rapid acting insulin such as lispro, aspart, or glulisine given directly after meals can be equally efficacious.

### 2.1. Does Glycemic Control Affect Outcome in Multiple Myeloma?

In a retrospective study done by Brunello et al. hyperglycemia correlated with nonhematological toxicity (neuropathy, fever, fatigue) in NHL patients [25]. Further studies are needed to assess the impact of hyperglycemia on hematological and nonhematological toxicity in patients with multiple myeloma. 

 Novel treatments in diabetes mellitus such as dipeptidyl peptidase IV (DPP4) inhibitors and Glucagon like peptide 1(GlP1) agonists can be theoretically used to control steroid-induced hyperglycemia or diabetes in MM; nevertheless, there are no studies till the present time that have looked into the effect of these new agents on cancer in general and multiple myeloma specifically. Some reports in the literature mentioned the possible adverse effects of DPP4 on parameters of immunity. Cells of the immune system such as thymocytes, T and B lymphocytes, and NK cells contain a cell surface protein called CD26; this latter has a DDP4 enzymatic activity and its activation was shown to increase the proliferation and/or activation of T cells and IL2 production. In addition, in vitro studies showed that DPP4 inhibitors modify T cell function by decreasing IL2, IL10, and interferon *γ* and increasing transforming growth factor *β*1 [26]. CD26 was also suggested to be implicated in autoimmunity and T cell response to external stimuli [26]. Likewise GLP1 receptor signaling was also found to regulate lymphocyte proliferation and maintenance of regulatory peripheral regulatory T cells in mice [27]. The effect of these novel antidiabetics on the immune system is still not apparent, and thus more research is needed on the use of such agents in patients with multiple myeloma and other lymphoproliferative disorders.

## 3. Thalidomide-Induced Hyperglycemia

In a study by Iqbal et al., thalidomide 150 mg or placebo was administered for 3 weeks in a crossover design to 6 patients with diabetes [28]. Insulin resistance was increased by 31% decreased insulin-stimulated peripheral glucose uptake, and glycogen synthesis was decreased by 48%; this was assessed by performing isoglycemic-hyperinsulinemic clamps before and after therapy. In another study by Wilson and Vallance-Owen [29], mothers giving birth to children with congenital malformations in 1966 were studied for insulin antagonism using a bioassay (rat diaphragm assay). 5 Out of 6 mothers (83%) exposed to thalidomide in their first trimester had antagonism to insulin whereas 14 out of 50 (28%) mothers in the control group had insulin antagonism. In 2001, Figg et al. showed that decreasing the dose of thalidomide improved hyperglycemia [30]. In 2003, a case report on thalidomide-induced severe hyperglycemia was published by Pathak et al. [31]. Overall, larger studies are needed to assess this risk and its implications on diabetes and multiple myeloma outcome.

## 4. Multiple Myeloma and Diabetic Neuropathy

Peripheral neuropathy is a common problem in patients with multiple myeloma and is also a common complication of type 2 diabetes. The condition may occur before initiating treatment [32]. In a recent study by Borrello et al., the incidence of peripheral neuropathy in patients with newly diagnosed multiple myeloma prior to the administration of any therapy was 15% which suggests that peripheral neuropathy is a symptom of the disease itself [33]. Furthermore treating multiple myeloma might complicate the neuropathy; the latter is associated with agents used to treat the disease, such as bortezomib [7], thalidomide [34], and vincristine [35]. Recently Wilson and Vallance-Owen have suggested an interaction between myeloma-related factors and the patient's genetic background in the development of treatment-induced peripheral neuropathy, with different molecular pathways being implicated in bortezomib-induced and vincristine-induced peripheral neuropathy [29]. Patients frequently complain of sensory symptoms, pain in a stocking-and-glove distribution, and proprioception changes that may affect normal daily living activities [36]. Studies looking into the association between bortezomib-induced neuropathy and diabetic neuropathy have yielded contradictory results. Badros et al. showed that the highest risk and grade of bortezomib neurotoxicity was observed in patients who had baseline peripheral neuropathy and diabetes mellitus [7]. In the APEX trial, more than 300 patients with refractory or relapsed multiple myeloma were randomized to bortezomib or dexamethasone. The investigators evaluated peripheral neuropathy. In this trial, the incidence and severity were not affected by age, number or type of prior therapies, baseline glycosylated hemoglobin level, or diabetes history [37]. Moreover the incidence of grade 3 peripheral neuropathy was actually lower in patients with a history of diabetes. The authors hypothesized that bortezomib-associated neuropathy is mechanistically distinct and that prior exposure to other neurotoxic agents or history of diabetes should not exclude patients from bortezomib therapy [37]. Finally in a more recent subanalysis of the phase 3, Vista trial that assed the frequency, characteristics, reversibility and prognostic factors for bortezomib associated peripheral neuropathy in newly diagnosed multiple myeloma patients ineligible for high-dose therapy who received bortezomib plus melphalan prednisone. Preexisting diabetes did not affect the overall rate of peripheral neuropathy whereas baseline neuropathy was the only consistent risk factor for any peripheral neuropathy (HR 1.785, *P* = 0.0065), grade ≥2 peripheral neuropathy (HR 2.205, *P* = 0.0032), and grade ≥3 peripheral neuropathy (HR 2.438, *P* = 0.023); moreover, bortezomib-associated peripheral neuropathy was reversible [31]. Vincristine, the oldest and most neurotoxic of the class, is still widely used in leukemias, lymphomas, myeloma, and various sarcomas. Peripheral neuropathy is the most common dose-limiting toxicity of vincristine. Symptoms range from peripheral sensorimotor loss to autonomic dysfunction related to paralytic ileus, orthostasis, and sphincter problems [28]. Thalidomide is an oral immunomodulatory and antiangiogenic agent. In the 1990s, it showed good results in multiple myeloma patients, and it received US Food and Drug Administration (FDA) approval in 1998. Thalidomide-induced peripheral neuropathy is characterized by being mainly distal sensory and less commonly motor. Its incidence varies from 25% to 75% [32]. The major predictors to thalidomide-induced peripheral neuropathy seem duration of treatment and possibly baseline neuropathy [38]. Peripheral neuropathy is a common complication of diabetes mellitus and multiple myeloma. Therefore, patients receiving a chemotherapeutic agent that might exacerbate peripheral neuropathy should be closely monitored. As for bortezomib-associated neuropathy, it was shown to be reversible in the majority of patients after dose reduction or discontinuation [6]. We suggest that newly diagnosed patients with multiple myeloma be clinically assessed for peripheral neuropathy prior to starting treatment and regularly assessed thereafter. The exact duration of posttreatment monitoring remains controversial and is dependent on diabetic history, baseline neuropathic symptoms, and the type and dose of chemotherapy received.

 Patients should also be educated about the symptoms to ensure early detection of neuropathy [38]. Stringent glycemic control may reduce the risk of developing diabetic neuropathy by 60% [23]. There are no consensus guidelines about diabetes management in multiple myeloma, but we can extrapolate from previous reports about diabetes management in cancer patients that first the progressive loss of nerve function associated with diabetic neuropathy can be slowed down by adequate glycemic control [25], and the latter is designated as the only modifiable risk factor for diabetic neuropathy [39]. The household environment should be adjusted to prevent falls, and water temperature should be decreased to prevent burns and use night lights. Proper foot and nail care should be emphasized to prevent ulcers and infection [40].

## 5. Multiple Myeloma and Nephropathy

Renal insufficiency is a common complication in patients with diabetes. It is also a common accompaniment of multiple myeloma. The presence of such complication in multiple myeloma patients along with diabetes creates an extra burden to the patient as well as the physician. It was reported that nephropathy is a poor prognostic indicator for survival in these two comorbid conditions [41].

Approximately, 20% of patients with newly diagnosed multiple myeloma can present with renal insufficiency, and up to 40% of patients with type 2 diabetes mellitus can be affected with diabetic nephropathy [42]. Nephropathy associated with multiple myeloma is usually due to abnormal light chains deposition. When this deposition is tubulopathic, it can lead to cast nephropathy in the distal tubules or more rarely Fanconi syndrome or type 2 renal tubular acidosis in the proximal tubules. Alternatively, when the deposition is glomerulopathic, it can lead to monoclonal immunoglobulin deposition disease or light chain amyloidosis [43, 44]. During the course of the multiple myeloma, approximately half of the patients will experience renal insufficiency either from the disease itself or as a complication of treatment [45]. The combination of new therapies for multiple myeloma causes rapid reductions of the monoclonal protein especially the free light chain which is the culprit for the cast nephropathy that is considered the most common renal lesion in multiple myeloma. Bortezomib and thalidomide are not cleared by the kidneys so they can be administered without dose adjustments in patients with renal failure. On the other hand, treatment with Lenalomide which is cleared renally requires careful creatinine monitoring and dose adjustments. Lenalomide has been shown to be efficacious and improved the kidney function in patients [46]. Dehydration, use of nonsteroidal anti-inflammatory drugs, hypercalcemia, and use of contrast agents are considered precipitating factors for renal failure in patients with concomitant diabetes and multiple myeloma. Special considerations should be taken in such patients. Since there are no reports that looked into this issue, it's of great importance to keep in mind that avoiding and treating the risks may ameliorate the severity of nephropathy, and adequate glycemic control may slow down the progression of diabetic nephropathy in these patients [47].

## 6. Multiple Myeloma and Retinopathy

The ocular manifestations can be the first presentation of the disease, and the mechanism includes direct infiltration or extramedullary plasmacytomas displacing surrounding tissues or by deposition of light chain in ocular tissues or by hyperviscosity state. The ophthalmic findings include proptosis, diplopia, lid ecchymosis, xanthomatosis, conjunctival and corneal crystalline and noncrystalline deposits, scleritis, episcleritis, secondary glaucoma, ciliary body cysts, ciliochoroidal effusion, uveal plasmacytoma, hyperviscosity retinopathy, retinal vasculitis, detachment of sensory retina and retinal pigment epithelium, and neuroophtalmic manifestations [48]. All These findings might complicate the diabetic retinopathy in patients with coexistent multiple myeloma and diabetes.

We suggest the all patients with multiple myeloma undergo ophthalmic evaluation at the time of diagnosis and be followed up closely by an ophthalmologist if baseline diabetic retinopathy was found. Additionally, strict glucose control is imperative in these patients.

## 7. Muliplte Myeloma and Cardiovascular Diseases

Diabetes is well known to be associated with increased risk of coronary heart disease and stroke [49]. Multiple myeloma can also possibly predispose to these 2 macrovascular complications. The presence of these conditions simultaneously worsens the prognosis and creates a bigger challenge to the treating physician.

Recently a case report about ischemic heart disease in a patient with multiple myeloma receiving bortezomib and dexamethasone has been published. The authors suggested that the mechanism could be explained by the inhibition of proteasome activity. This inhibition increases endothelial progenitor cell apoptosis [50] and decreases its proliferation which affects endothelial nitric oxide synthase/nitric oxide leading to coronary spam [51–53]. Moreover, an age-dependent decrease in ubiquitin-proteasome activity has been associated with injury of heart muscles and morbidity of cardiovascular diseases. The bortezomib-induced decrease in proteasome activity has been linked to increased rate of apoptosis in smooth muscle cells [54], thus, causing a weakening of the fibrous cap and eventually leading to atherosclerotic plaque instability and rupture [55–57]. Moreover, multiple myeloma has been associated with cardiac amyloidosis which can exacerbate the heart failure that might already be present in patients with diabetes mellitus [58].

Stroke can be a complication of multiple myeloma as part of the hyperviscosity syndrome associated with the disease due to the paraproteinemia [59]. This might be an added risk to patients with diabetes mellitus who already have an increased risk.

## 8. Conclusion

Diabetics with multiple myeloma constitute a challenging specific population to physicians. Multiple myeloma by itself and its related treatments can complicate the microvascular and macrovascular complications of diabetes. The treating physician has to recognize the treatment-related complications and closely follow up diabetic patients for the emergence or the worsening of hyperglycemia, neuropathy, nephropathy, or retinopathy in addition to cardiovascular diseases. In addition, maintaining adequate blood glucose levels reduces the risk of infection in patients with multiple myeloma and decreases the risk and severity of diabetic microvascular complications, thus, minimizing the increased morbidity of multiple myeloma [60].
